# Supplementary material for: Drug repurposing for aging research using model organisms
Source: Aging Cell. 2017 Jun 16;16(5):1006–15. doi: 10.1111/acel.12626 (PMC5595691; doi:10.1111/acel.12626)
Supplement: Supplementary file 7 — Data S1 Zip‐Archive of all report cards. [file ACEL-16-1006-s007.zip › RC_AMZ.pdf]

AMZ

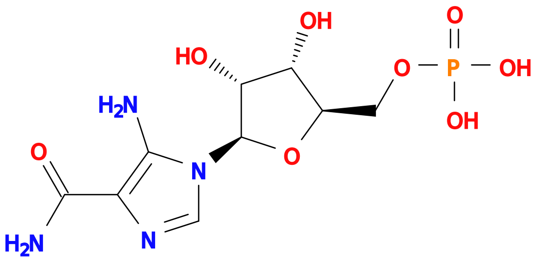

Database identifiers

ChEMBLCompound	CHEMBL483849
CHEBI	18406
eMolecules	6880888

Ranking

	Rank	Score
Drosophila	494/697	0.283
C. elegans	515/591	0.057

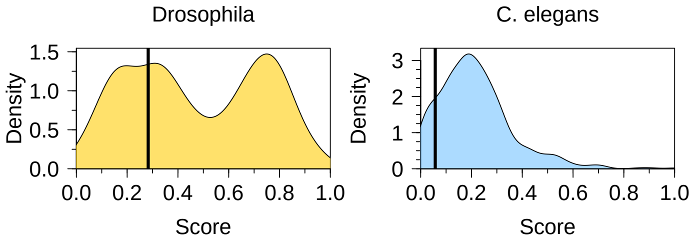

	Ageing implication	Domain conservation	Binding site conservation	Binding affinity	Bioavailability	Lipinski	Promiscuity	Purchasability	Drug approval	**Total**
Drosophila	0.81	0.948	0.9	0.456	(0.9)	-0.1	-0.0	0.1	0.0	0.283
C. elegans	0.81	0.922	0.83	0.456	0.205	-0.1	-0.0	0.1	0.0	0.057

Names

- **AICA ribonucleotide**
- 5'-Phospho-ribosyl-5-amino-4-imidazole carboxamide
- 5-Amino-1-(5-phospho-D-ribosyl)imidazole-4-carboxamide
- 5-aminoimidazole-4-carboxamide ribotide
- 5-Phosphoribosyl-4-carbamoyl-5-aminoimidazole
- acadesine 5'-monophosphate
- AICAR

Roles

plant metabolite, human metabolite, Escherichia coli metabolite, Saccharomyces cerevisiae metabolite, cardiovascular drug

Status

Approved drug (according to ChEMBL)	No
Number of Rule of 5 violations	2
Binding affinity to original target in log units (RF-Score prediction)	4.82
Burns *C. elegans* bioavailability prediction	-7.41

Compound Target Characteristics

5'-AMP-activated protein kinase subunit gamma-1

Best gene implication in ageing for this target family came from gene P12904 annotated in UniProt release 2014_02. Annotation GO subterm of 7568 (aging) was Inferred from Mutant Phenotype

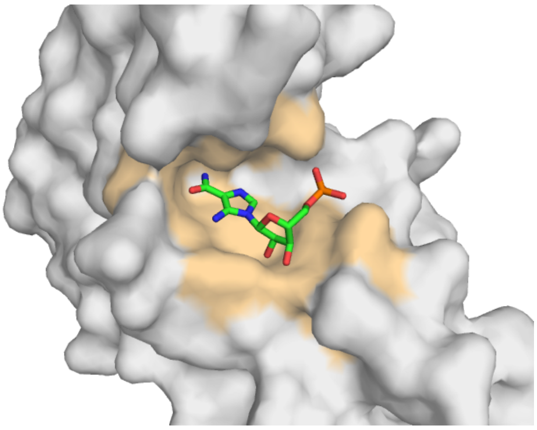

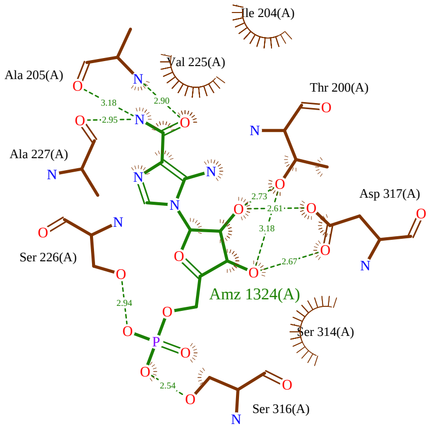

protein	amino acids contacts (binding site)									
PDB:2uv5:chainA:P54619	T	I	A	V	S	A	I	S	S	D
tr:F8W9D1:F8W9D1_HUMAN	T	I	A	V	S	A	I	S	S	D
tr:F8VYY9:F8VYY9_HUMAN	T	I	A	V	S	A	I	S	S	D
sp:P54619:AAKG1_HUMAN	T	I	A	V	S	A	I	S	S	D
sp:P80385:AAKG1_RAT	T	I	A	V	S	A	I	S	S	D
sp:O54950:AAKG1_MOUSE	T	I	A	V	S	A	I	S	S	D
tr:Q3TWR3:Q3TWR3_MOUSE	T	I	A	V	S	A	I	S	S	D
tr:Q7KNS9:Q7KNS9_DROME	T	I	E	V	S	A	I	S	S	D
tr:Q8IGX0:Q8IGX0_DROME	T	I	E	V	S	A	I	S	S	D
tr:Q0KI34:Q0KI34_DROME	T	I	E	V	S	A	I	S	S	D
tr:A1A703:A1A703_DROME	T	I	E	V	S	A	I	S	S	D
tr:A1A6Y3:A1A6Y3_DROME	T	I	E	V	S	A	I	S	S	D
tr:O96613:O96613_DROME	T	I	E	V	S	A	I	S	S	D
tr:Q8I0K6:Q8I0K6_DROME	T	I	E	V	S	A	I	S	S	D
tr:Q8I0A1:Q8I0A1_DROME	T	I	E	V	S	A	I	S	S	D
tr:Q8SXT8:Q8SXT8_DROME	T	I	E	V	S	A	I	S	S	D
tr:Q8I0B2:Q8I0B2_DROME	T	I	E	V	S	A	I	S	S	D
tr:Q7JQX9:Q7JQX9_DROME	T	I	E	V	S	A	I	S	S	D
tr:Q9VDD2:Q9VDD2_DROME	T	I	E	V	S	A	I	S	S	D
tr:Q9BHL6:Q9BHL6_CAEEL	A	I	L	V	S	A	V	S	S	D
sp:P12904:AAKG_YEAST	T	M	K	V	S	S	V	T	S	D
tr:F4ZZV2:F4ZZV2_YEASX	T	M	K	V	S	S	V	T	S	D

protein	whole protein ident	simil	domain-based ident	simil	contact-based ident	simil
PDB:2uv5:chainA:P54619	0.99	1.0	1.0	1.0	1.0	1.0
tr:F8W9D1:F8W9D1_HUMAN	0.75	0.75	1.0	1.0	1.0	1.0
tr:F8VYY9:F8VYY9_HUMAN	0.84	0.84	1.0	1.0	1.0	1.0
sp:P54619:AAKG1_HUMAN	1.0	1.0	1.0	1.0	1.0	1.0
sp:P80385:AAKG1_RAT	0.96	0.99	0.99	1.0	1.0	1.0
sp:O54950:AAKG1_MOUSE	0.97	0.99	0.99	1.0	1.0	1.0
tr:Q3TWR3:Q3TWR3_MOUSE	0.97	0.99	0.99	1.0	1.0	1.0
tr:Q7KNS9:Q7KNS9_DROME	0.39	0.54	0.66	0.89	0.9	0.9
tr:Q8IGX0:Q8IGX0_DROME	0.37	0.53	0.66	0.89	0.9	0.9
tr:Q0KI34:Q0KI34_DROME	0.37	0.53	0.66	0.89	0.9	0.9
tr:A1A703:A1A703_DROME	0.33	0.46	0.66	0.89	0.9	0.9
tr:A1A6Y3:A1A6Y3_DROME	0.32	0.46	0.65	0.89	0.9	0.9
tr:O96613:O96613_DROME	0.31	0.44	0.66	0.89	0.9	0.9
tr:Q8I0K6:Q8I0K6_DROME	0.28	0.4	0.66	0.89	0.9	0.9
tr:Q8I0A1:Q8I0A1_DROME	0.25	0.35	0.66	0.89	0.9	0.9
tr:Q8SXT8:Q8SXT8_DROME	0.22	0.31	0.66	0.89	0.9	0.9
tr:Q8I0B2:Q8I0B2_DROME	0.22	0.31	0.66	0.89	0.9	0.9
tr:Q7JQX9:Q7JQX9_DROME	0.21	0.3	0.66	0.89	0.9	0.9
tr:Q9VDD2:Q9VDD2_DROME	0.14	0.2	0.66	0.89	0.9	0.9
tr:Q9BHL6:Q9BHL6_CAEEL	0.28	0.44	0.54	0.85	0.7	0.83
sp:P12904:AAKG_YEAST	0.3	0.71	0.38	0.81	0.5	0.72
tr:F4ZZV2:F4ZZV2_YEASX	0.3	0.71	0.38	0.81	0.5	0.72

SNF4Agamma (FBgn0264357) associated phenotypes

feeding behavior defective, hyperactive, neuroanatomy defective, nutrition conditional, phototaxis defective, short lived, starvation stress response defective

(Information from FlyBase)
